# Cutaneous *Mycobacterium chelonae* infection presenting clinically as a mycetoma

**DOI:** 10.1016/j.jdcr.2021.11.001

**Published:** 2021-11-16

**Authors:** Katie Dreher, Amanda Ederle, Eric Rosenbaum, Rodrigo Valdes-Rodriguez

**Affiliations:** a College of Medicine, University of Arkansas for Medical Sciences, Little Rock, Arkansas; b Baptist Health Medical Center, North Little Rock, Arkansas; c Department of Pathology, University of Arkansas for Medical Sciences, Little Rock, Arkansas; d Department of Dermatology, University of Florida Health, Gainesville, Florida

**Keywords:** infectious disease, mycetoma, mycobacteria, *Mycobacterium chelonae*, nontuberculous mycobacteria, rapidly growing, NTM, nontuberculous mycobacteria, AFB, acid-fast bacilli

## Introduction

*Mycobacterium chelonae* belongs to a group of rapidly growing mycobacteria, a subset of the ubiquitous nontuberculous mycobacteria (NTM) that rarely cause skin and soft–tissue infections.^1^ These infections often occur in immunosuppressed patients but may also be seen in immunocompetent hosts.^1^^,^^2^ Their incidence is increasing worldwide and is thought to result from a combination of more surgical and cosmetic procedures performed, the increased use of immunosuppressive medications, and an improved ability to isolate and identify these organisms.^1^^,^^3^^,^^4^ Misdiagnoses and delayed diagnoses are still common despite this increased incidence,^2^^,^^5^ thus making the ability to recognize unusual clinical presentations of cutaneous NTM infections increasingly important.

## Case report

A 54-year-old man with a past medical history significant for end–stage renal disease secondary to diabetes mellitus presented to the clinic with a 3-month history of nonhealing, painless, ulcerated nodules on his lower leg. The patient had been hospitalized 5 months prior for sepsis and acute hypoxic respiratory failure secondary to SARS-CoV-2 infection, during which he received high-dose methylprednisolone for 19 days. Two months later, he was hospitalized for *Enterococcus faecalis* endocarditis, during which his lower-extremity lesions were first noted. Upon further questioning, the patient reported frequent outdoor activity, including fishing and gardening. He denied any barefoot walking, trauma to the affected area, or recent travel.

**Fig 1:**
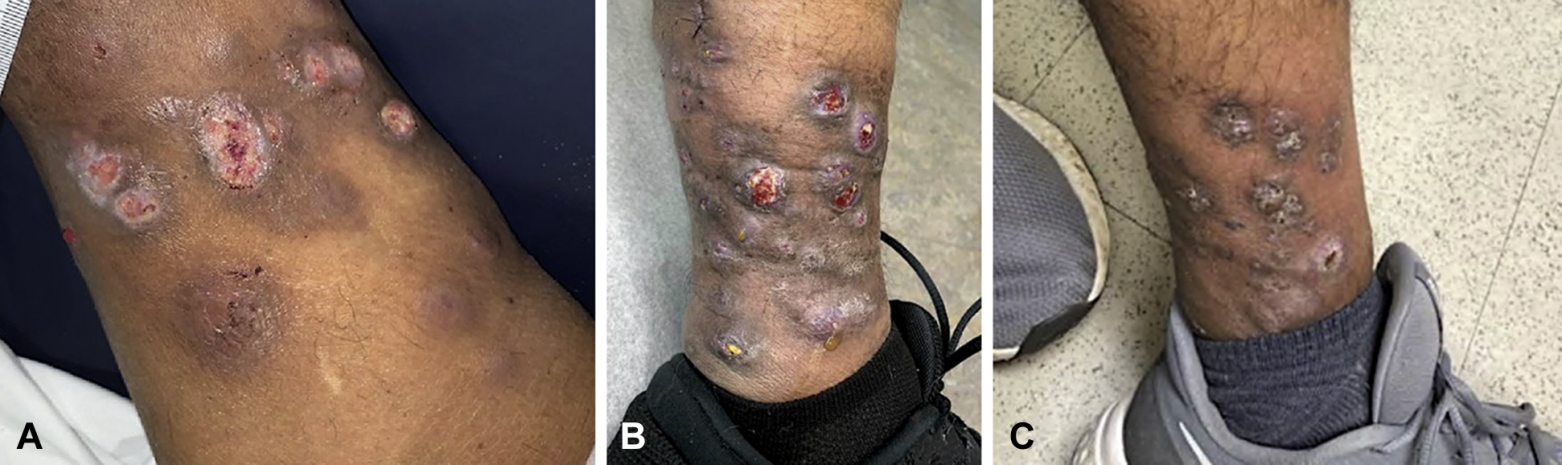
Cutaneouos *Mycobacterium chelonae* infection. **A**, Initial clinical presentation showing violaceous, friable, ulcerated nodules scattered on the left medial lower extremity. **B**, Clinical image taken at 2-month follow-up showing worsening of lesions. There are multiple, grouped, erythematous nodules on the left medial lower extremity with ulceration, purulent crusting, and sinus tract formation. **C**, Improvement of patient's lesions seen after 3 months of combined antibiotic therapy with tobramycin, linezolid, and azithromycin.

**Fig 2:**
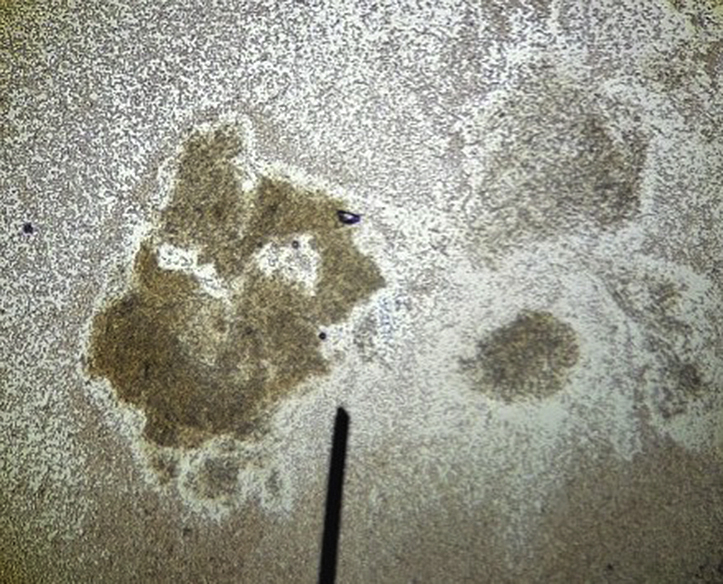
Dark brown granule seen on wet preparation of purulence aspirated from one of the patient's draining sinus tracts.

A skin examination was significant for violaceous, friable, ulcerated nodules scattered on the left medial lower extremity (Fig 1, *A*). A punch biopsy demonstrated a nonspecific, mixed inflammatory infiltrate in addition to scattered fibrosis and necrosis. Tissue Gram, periodic acid–Schiff, Fite, and acid-fast bacilli (AFB) stains were all negative. A skin examination at a 2-month follow-up revealed worsening of the lesions, with multiple grouped, erythematous nodules on a background of hyperpigmentation and edema (Fig 1, *B*, left lower medial leg). Many nodules showed ulceration with purulent crust and draining sinus tract formation. The purulent material was aspirated and examined by wet preparation and showed dark brown grains (Fig 2), raising suspicion for mycetoma, despite its rarity in the United States.^6^ An excisional biopsy of one of the nodules was performed.

**Fig 3:**
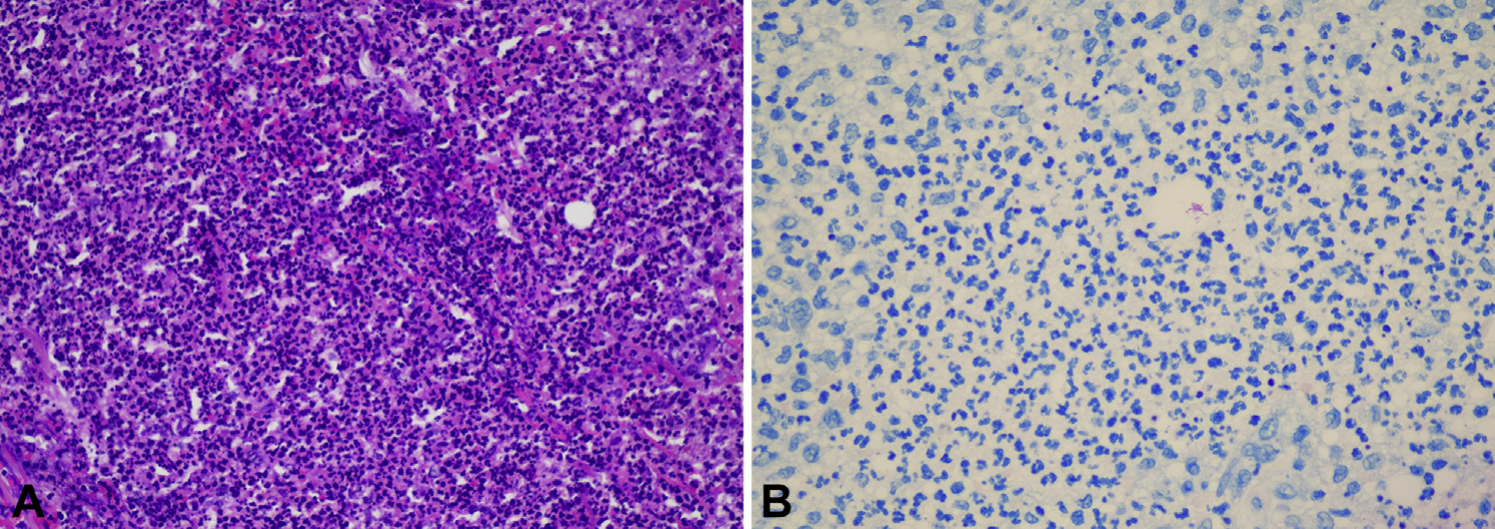
Cutaneous *Mycobacterium chelonae* infection. **A**, Neutrophilic dermal inflammation intermixed with scattered lymphocytes and histiocytes (hematoxylin-eosin stain). **B**, Acid-fast bacilli apparent on acid-fast bacilli staining.

**Fig 4:**
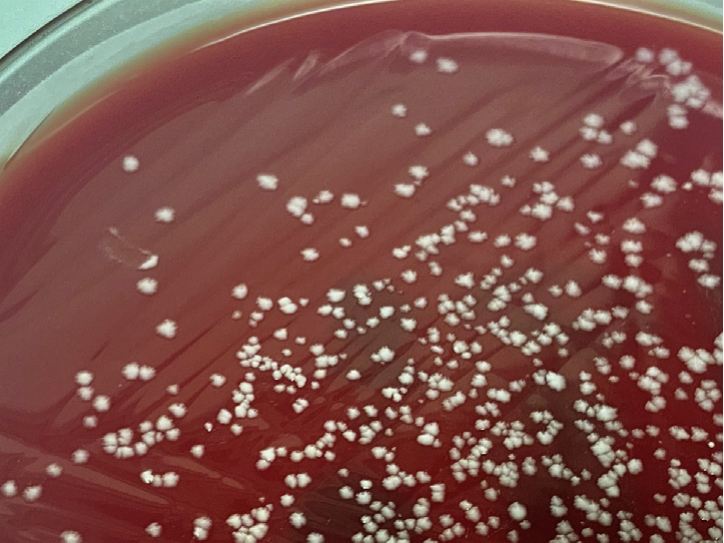
Mycobacterial colonies growing on the blood agar plate used for acid-fast bacilli tissue culture. *Mycobacterium chelonae* was identified via mass spectrometry.

Histopathology showed exuberant, neutrophilic dermal inflammation intermixed with scattered lymphocytes and histiocytes (Fig 3, *A*). The AFB stain revealed a few AFB (Fig 3, *B*), while the tissue Gram and periodic acid–Schiff stains failed to highlight any fungal or bacterial elements. Despite examining multiple levels of tissue, no grains characteristic of mycetoma were found. Fresh tissue submitted for culture grew *M. chelonae* (Fig 4). The fungal culture was negative, and the other bacteria comprised only normal skin flora. A repeat tissue culture confirmed these results.

Based on susceptibility testing, the patient was treated with 6 months of oral azithromycin (500 mg, daily) and linezolid (600 mg, twice daily). He was also treated with 3 months of intravenous tobramycin (200 mg, 3 times weekly for 1 month, followed by 320 mg, once weekly for 2 months, based on tobramycin trough and peak monitoring). He will continue azithromycin and linezolid for 2 additional months, for a total of 8 months (or longer, if clinically necessary). The patient's lesions have improved significantly with therapy (Fig 1, *C*).

## Discussion

This patient exhibited the clinical triad of mycetoma: painless, subcutaneous masses, the formation of multiple sinus tracts, and grains identified from purulent discharge.^7^^,^^8^ The presence of the lesions on the lower extremity, where mycetoma is located in over 80% of cases, further increased our suspicion for this condition.^7^^,^^8^ To our knowledge, mycobacterial species have never been reported as isolated causes of cutaneous mycetoma. The one case report identifying M. chelonae in a mycetoma also noted the presence of *Exophiala jeanselmei*, a fungus known to cause mycetoma.^9^ The absence of organisms characteristic of mycetoma identified in our patient precluded us from making this diagnosis based on the current understanding of the disease; however, the infection's clinical resemblance to mycetoma was striking. Due to the utilization of AFB staining and culture, the correct diagnosis of a cutaneous NTM infection was eventually reached. However, the diagnosis and treatment were delayed due to repeat culture in an attempt to identify the expected fungi or filamentous bacteria, as *M. chelonae* is not typically associated with grain formation.^7^

Cutaneous infections with NTM have a widely variable and often nonspecific clinical appearance.^1^^,^^2^ *M. chelonae* infections commonly present as disseminated skin lesions,^5^ though they may also appear as localized cellulitis, abscesses, ulcers, subcutaneous nodules, or, occasionally, the sinus tract formation seen in our patient.^1^ Due to this lack of specificity, high clinical suspicion with testing specific for mycobacteria is critical for an accurate diagnosis. This is true even when lesions closely mimic other cutaneous infections, especially if the lesions are chronic and prior treatments have been unsuccessful.^1^^,^^2^^,^^5^

The diagnosis may be hindered by the low sensitivity of common mycobacterial identification methods in NTM infections. One study showed that in patients with positive AFB cultures, only 15% and 34.2% had positive AFB smears and Fite stains, respectively.^2^ This low sensitivity was also evidenced in our patient, whose AFB and Fite stains were negative on the initial biopsy. In the presence of reasonable clinical suspicion, an AFB culture should be strongly considered, even when stains are negative.

In addition to overlapping clinical features, NTM skin infections and mycetomas share several risk factors. As with organisms known to cause mycetoma, NTM species are often isolated from environmental sources, such as freshwater, soil, and plant matter.^5^ Since our patient was an avid fisherman and gardener, exposure to both groups of pathogens was possible. Furthermore, both subsets of organisms are also frequently introduced by trauma.^1^^,^^7^ Although our patient could not recall any trauma to the area, mild, unnoticed trauma could not be ruled out due to the prolonged course of his infection in the setting of diabetic peripheral neuropathy. An immunosuppressed state has been recognized as both a risk factor and a cause of more severe infections in both NTM infections and some subtypes of mycetoma.^3^^,^^7^^,^^8^ Our patient's recent, prolonged course of high-dose corticosteroids, end–stage renal disease, and diabetes mellitus may have increased his susceptibility.^2^, ^3^, ^4^ Unlike mycetoma, a *M. chelonae* infection has also been associated with surgical and cosmetic procedures, including Mohs surgery, botulinum toxin injection, and tattooing.^1^^,^^4^^,^^5^ The presence of these factors should be assessed to risk-stratify patients.

## Conclusion

Despite an increasing incidence of cutaneous NTM infections, incorrect or delayed diagnoses are still common.^4^^,^^5^ These infections' abilities to closely mimic other cutaneous infections, challenges in identification, and overlapping risk factors with other microorganisms may all play a role.^2^^,^^7^ Additional studies and case reports of cutaneous, rapidly growing NTM infections are necessary to promote awareness and the timely diagnosis of this condition.^5^ The ability of *M. chelonae* to form granules clinically, though not a classically reported feature, should also be considered.

## Conflicts of interest

None disclosed.
